# Special Chimeric Antigen Receptor (CAR) Modifications of T Cells: A Review

**DOI:** 10.3389/fonc.2022.832765

**Published:** 2022-03-22

**Authors:** Lele Miao, Juan Zhang, Binjie Huang, Zhengchao Zhang, Song Wang, Futian Tang, Muzhou Teng, Yumin Li

**Affiliations:** ^1^ Department of General Surgery, Second Hospital of Lanzhou University, Lanzhou, China; ^2^ Key Laboratory of the Digestive System Tumors of Gansu Province, Lanzhou, China; ^3^ Department of Hematology, Fifth Medical Center, Chinese PLA General Hospital, Beijing, China; ^4^ Lanzhou University, Lanzhou, China

**Keywords:** special CARs, improve, efficacy, safety, conjectures

## Abstract

Chimeric antigen receptor (CAR) -T cell therapy has become one of the hot topics in tumor immunity research in recent years. Although CAR-T cell therapy is highly effective in treating hematological malignancies, there are numerous obstacles that prevent CAR-T cells from having anti-tumor effects. Traditional CARs, from the first to the fourth generation, are incapable of completely overcoming these challenges. Therefore, identifying ways to boost the efficacy of CAR-T cells by utilizing the limited tumor surface antigens has become an urgent area of research. Certain special CARs that have special structures, special systems, or are greatly improved on the basis of traditional CARs, such as tandem CAR, dual-signaling CARs, AND-gate CARs, inhibitory CAR, AND-NOT CARs, CARs with three scFvs, ON/OFF-switch CARs, and universal CARs have been introduced. This study aims to use these special CARs to improve the anti-tumor ability, accuracy, and safety of CAR-T cells. In addition to summarizing various special CARs of T cells, this paper also expounds some of our own conjectures, aiming to provide reference and inspiration for CARs researchers.

## Introduction

CAR-T cell therapy is a type of tumor immunotherapy that has developed rapidly in recent years. Currently, CAR-T cell therapy is mainly used to treat hematological malignancies, and it has had considerable success. However, the biological characteristics of solid tumors are more complex, posing many obstacles to CAR-T cells (1–4), including CAR-T cells homing barriers, tumor microenvironment (TME) inhibition, CAR-T cells trogocytosis (5), tumor antigen heterogeneity (6), CAR-T cells toxic responses, among others. These obstacles inevitably influence the anti-tumor effects of CAR-T cells in solid tumors. At present, CAR has developed from the first generation to the fifth generation. The intracellular structure of first-generation CARs only has one signal structure domain. The second-generation CARs add one co-stimulatory molecule to the first-generation CAR. The third-generation CARs add 2 costimulatory molecules. The fourth-generation CARs are modified by adding the cytokine inducer or suicide genes based on the second-generation or the third-generation CARs. The fifth-generation CARs added a “third party” intermediate system in the extracellular domain. None of the traditional CARs, from the first-generation to the fourth-generation, can completely overcome the aforementioned obstacles. Nowadays, in addition to searching for tumor-specific antigens (TSAs), constructing new special CARs or optimizing traditional CARs to minimize their toxicity and kill tumor cells more efficiently has become the main research focus of CAR-T cell therapy. This review will be elaborated from the following four aspects, including optimizing the recognition ability of CARs, improving the accuracy of CARs, improving the killing ability of CARs and improving the safety of CARs.

## Optimize the Recognition Ability of CARs

### Double scFvs

Double scFvs are designed with two corresponding scFvs for two different tumor surface antigens. Examples of CARs that used double scFvs include “tandem CARs”, “dual-signaling CARs”, “AND-gate CARs”, and “inhibitory CARs”.

#### The Tandem CAR (TanCAR)

**Figure 1:**
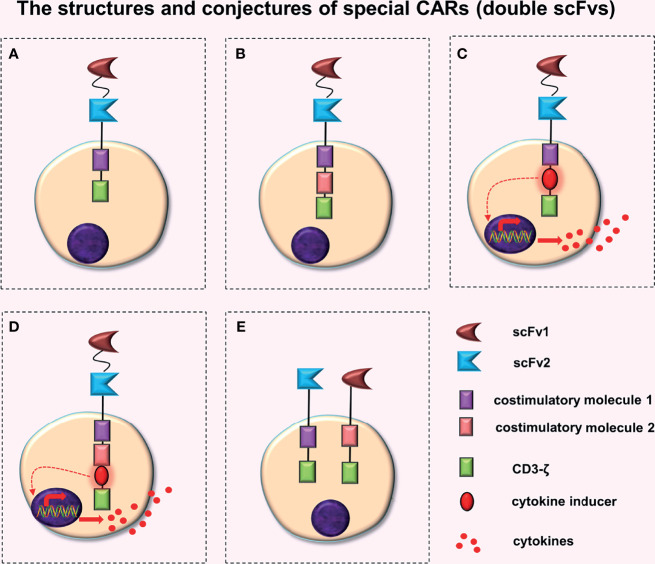
The structures and conjectures of special CARs (double scFvs). Construction of a TanCAR based on the **(A)** second-generation CAR, **(B)** third-generation CAR, **(C)** fourth-generation CAR (the intracellular domain contains a costimulatory molecule), and **(D)** fourth-generation CAR (the intracellular domain contains two costimulatory molecules). **(E)** Construction of the dual-signaling CARs based on the second-generation CAR.

TanCAR adopts a design concept of the “OR” gate. Two different scFvs are connected in the extracellular domain of a CAR. Grada et al. (7) constructed TanCAR using gene-editing technology to connect two different series of scFvs to a single transgenic receptor. The two scFvs of TanCAR were connected outside the cell (in series) by a Gly-Ser linker and had good flexibility (**Figure 1A**) . The TanCAR, which is activated when any one of the scFvs binds to a target antigen, can enable a CAR-T cell to synchronously recognize two types of tumor surface antigens. When two scFvs bind to their respective target antigens, the TanCAR will not only be activated but will also produce synergistic effects to further improve the activation of CAR-T cells and their tumor killing ability (7–9). The synergistic effect may be caused by binding two or more antigens simultaneously, which may enhance the signal transduction of immune synapses (10). When compared to traditional CARs with only one scFv, TanCAR-T cells have a higher anti-tumor effect and can limit tumor cell immune evasion (10–13).

A plasmid is very small, which makes it impossible to add gene elements to it without restriction. Therefore, Grada et al. (7) constructed the TanCAR based on a second-generation CAR, whereas Zhao et al. (14) successfully constructed a TanCAR using human trophoblast cell surface antigen 2 (Trop 2) and programmed death-ligand 1 (PD-L1) as targets, based on the third-generation CAR (**Figure 1B**). Preclinical experiments revealed that (Trop2/PD-L1)-CAR-T cells significantly enhanced the killing effect on gastric cancer cells when compared to Trop2-CAR-T cells or PD-L1-CAR-T cells.

### Conjectures

A TanCAR constructed based on the fourth-generation CAR, i.e. a TanCAR that has the function of a fourth generation CAR, can secrete additional anti-tumor cytokines when activated. Extracellular domains of these CARs have an extra scFv compared to traditional fourth-generation CARs (**Figures 1C, D**). These two designs (**Figures 1C, D**) will further complicate plasmid construction. These designs may increase the antigen recognition range of CAR-T cells and may produce synergistic effects (7–9), which will enhance the anti-tumor activity of CAR-T cells. Although no relevant literature has been retrieved, the structure and principle of these two CAR-T cells are relatively simple, indicating that they may already exist or are being developed.

#### Dual-Signaling CARs

Dual-signaling CARs refer to the expression of two separate CARs on the same T cell respectively and simultaneously, with each CAR cell having its own intracellular domains (**Figure 1E**). Ruella et al. (15) constructed dual-signaling CARs (targeting CD19 and CD123) by first creating two kinds of plasmids (CD19-CAR and CD123-CAR) respectively, and then transfecting the same T cell successively with lentivirus one by one. Finally, dual-signaling CAR-T cells, which express two types of CARs simultaneously, were screened out. Preclinical trials indicated that dual-signaling CAR-T cells had stronger anti-tumor activity than single-expression of CAR-T cells (CD19-CAR-T cells or CD123-CAR-T cells) or the mixed combination of CAR-T cells (CD19-CAR-T cells and CD123-CAR-T cells), and could better prevent disease recurrence caused by downregulation or loss of target antigens on the tumor cell surface.

### Triple scFvs (Arranged in Tandem)

**Figure 2:**
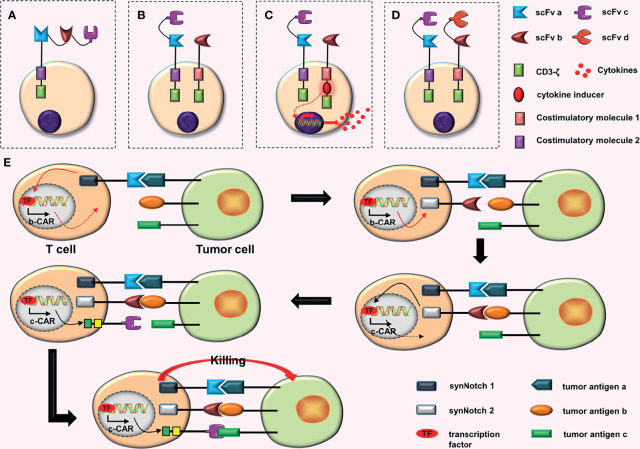
Some conjectures about CARs containing three scFvs. **(A)** Connecting three scFvs in tandem. **(B)** In dual-signaling CARs, one CAR is replaced by a TanCAR. **(C)** One of the dual-signaling CARs is replaced with a TanCAR, and the other is upgraded to a fourth-generation CAR. **(D)** Both CARs in dual-signaling CAR are replaced with tandem CARs. **(E)** At first, synNotch 1 receptor is activated after binding to tumor antigen (a), and the activated synNotch 1 receptor induces the expression of synNotch 2 receptor. Then synNotch 2 receptor binds to tumor antigen (b), which induces the expression of c-CAR; c-CAR can bind to the tumor antigen (c); finally, the CAR-T cell is activated.

The antigen binding domain of CAR is composed of three scFvs in tandem (**Figure 2A**). Bielamowicz et al. (16) created a trivalent-tandem CAR with a single universal tricistronic transgene to treat glioblastoma. The trivalent-tandem CAR could target and recognize three different TAAs on the surface of glioblastoma cells, including human epidermal growth factor receptor 2 (HER2), interleukin-13 receptor subunit alpha-2 (IL13Rα2), and ephrin-A2 (EphA2). The results showed that trivalent-tandem CAR-T cells have greater anti-tumor activity and can overcome tumor antigen heterogeneity than nonspecific or bispecific CAR-T cells. Balakrishnan et al. (17) also conducted a similar study, constructing a trivalent-tandem CAR by connecting three scFvs in tandem with designed ankyrin repeat proteins (DARPins). The results demonstrated that these CAR-T cells have potent anti-tumor effects and can better cope with tumor antigen heterogeneity and immune escape.

The trivalent-tandem CARs were constructed by Bielamowicz et al. (16) and Balakrishnan et al. (17) using intracellular costimulatory molecules CD28 and 4-1BB, respectively, based on second-generation CARs. Because it has been extremely difficult to build a trivalent-tandem CAR, making additional improvements based on it, such as adding a costimulatory molecule or constructing a tetravalent-tandem CAR, is more challenging. The vector volume is also extremely high, hindering the transmission of efficient genes, and the subsequent transfection rate may be very low. Furthermore, determining whether the genes can be successfully translated into proteins is a great challenge.

### Conjectures

The following three combinations can be used in combining dual-signaling CARs with the TanCAR.

a. Replacing one of the CARs in dual-signaling CARs with a TanCAR (**Figure 2B**). This design will increase the recognition range of CAR-T cells for targets to three different TAAs.
b. Replacing one CAR among the dual-signaling CARs with a TanCAR, and upgrading the other to a fourth-generation CAR (**Figure 2C**). This design will increase the recognition range of CAR-T cells for targets, which can recognize three different TAAs. Furthermore, the inclusion of four-generation CAR may further enhance anti-tumor effects of CAR-T cells.
c. Replacing both CARs in dual-signaling CAR with TanCARs (**Figure 2D**). This design will further increase the recognition range of CAR-T cells for targets, resulting in the recognition of four different TAAs.

The advantage of these CAR-T cells is that they can be activated when one scFv binds to the corresponding tumor antigen. When all scFvs bind to the corresponding tumor antigens, they may have synergistic effects, enhancing the anti-tumor ability of CAR-T cells (7–9). Moreover, certain designs, such as trivalent-tandem CARs, can effectively cope with tumor antigen heterogeneity and immune escape (11, 17–19).

## Improving the Accuracy of CARs

### AND-gate CARs

**Figure 3:**
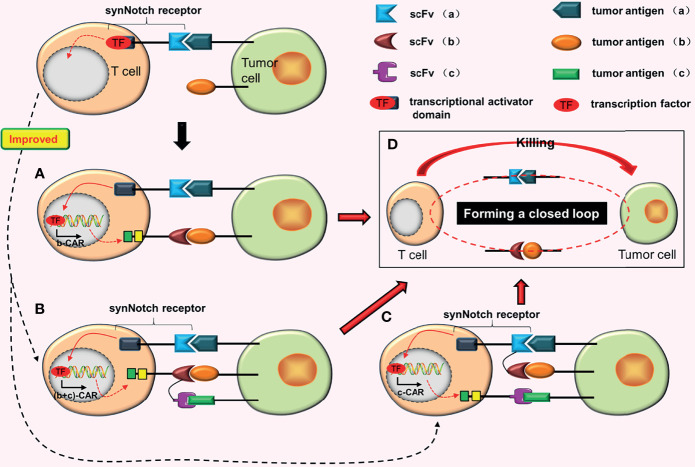
AND gate CARs. **(A)** When the synNotch receptor binds to tumor antigen (a), the transcriptional activator domain of the receptor is released, which can enter the nucleus and drive the expression of b-CAR genes. Subsequently, b-CAR will be expressed on the surface of the T cell, and the newly expressed b-CAR specifically binds to tumor antigen (b) to form a closed loop. The CAR-T cell is finally activated. **(B)** The synNotch receptor binds to tumor antigen (a) and induces TanCAR (b + c) expression. At this point, as long as one or two scFvs of this TanCAR bind to the corresponding tumor antigens, the T cell will be activated. **(C)** Adding a TanCAR to the synNotch receptor. As long as one of the scFv or two scFvs of this TanCAR bind to the corresponding tumor antigens, the expression of c-CAR will be induced. **(D)** Once the closed loop is formed, CAR-T cells will be activated.

AND-gate CARs adopt the “AND” gate design concept, and can only be activated when two scFvs bind to corresponding tumor antigens simultaneously. Roybal et al. (20) invented the synNotch receptor (a novel modular receptor), which is the core design of AND-gate CARs. The synNotch receptor binds to tumor antigen (a), inducing the expression of b-CAR, which selectively binds to tumor antigen (b). Finally, the T cell is activated (**Figure 3A**). SynNotch AND-gate T cells can only kill tumor cells that express double target antigens. Consequently, various studies (21–24) have proved that SynNotch AND-gate T cells are considerably safe and accurate, because they are ineffective against tumor cells expressing a single antigen while efficient against tumor cells expressing double target antigens.

The purpose of synNotch AND-gate CARs is to improve the precision of CAR-T cells. To further improve the antigen recognition range and anti-tumor effects of synNotch AND-gate CAR-T cells, Sabahi et al. (25) proposed the construction of tandem AND-gate CARs by combining TanCAR with AND-gate CARs. Tandem AND-gate CARs are activated as follows: the scFv (a) on the synNotch receptor induces the tandem CAR (b + c) after binding with tumor antigen (a) and the T cell is activated soon after as long as one scFv of the TanCAR is attached to the target antigen (**Figure 3B**). Recently, Williams et al. (26) successfully constructed the tandem AND-gate CARs. The experimental results showed that these CAR-T cells not only have high accuracy, but also have the potential to increase the recognition range of CAR-T cells and improve their ability to kill tumor cells. Furthermore, they linked the synNotch receptor with a TanCAR to construct another type of AND-gate CARs (26). The formation of c-CAR is induced as long as one scFv of the TanCAR is attached to the corresponding tumor antigen (**Figure 3C**). CAR-T cells with this structure are more easily activated.

### Conjectures

**Figure 4:**
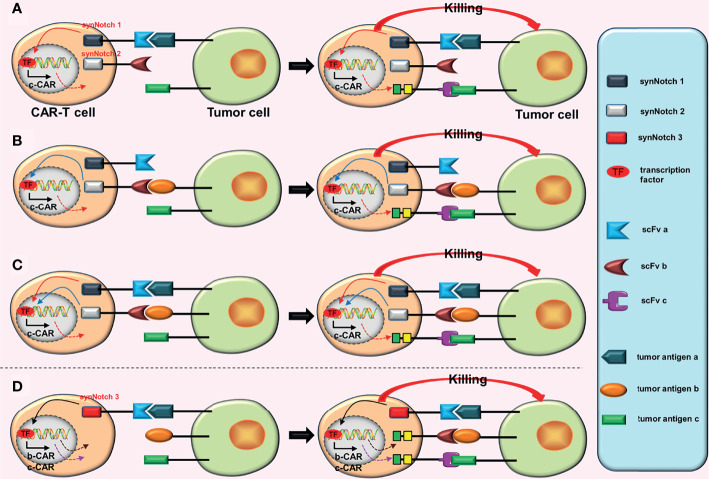
Some conjectures on the construction of “dual-signaling CARs” combined with “AND gate CARs”. **(A)** a-CAR of dual-signaling CARs binds to the corresponding tumor antigen (a), which can induce the expression of c-CAR. After c-CAR binds to tumor antigen (c), the CAR-T cell will be activated. **(B)** b-CAR of dual-signaling CARs binds to the corresponding tumor antigen (b), which can induce the expression of c-CAR. After c-CAR binds to tumor antigen (c), the CAR-T cell will be activated. **(C)** The expression of c-CAR can be induced by the combination of a-CAR and b-CAR with corresponding tumor antigens. After c-CAR binds to tumor antigen (c), the CAR-T cell will be activated. **(D)** The combination of a-CAR with corresponding tumor antigen (a) can induce the expression of b-CAR and c-CAR. As long as one of b-CAR and c-CAR binds to the corresponding tumor antigen, the CAR-T cell will be activated.

a. Combining dual-signaling CARs with AND-gate CARs may have two forms of construction. In the first structural form, two CARs in dual-signaling CARs add a synNotch receptor in the intracellular domain; as long as one of the CARs is attached to the target antigen, the formation of the third CAR will be induced (**Figures 4A, B**). Furthermore, two CARs combine with target antigens while inducing the formation of the third CAR (**Figure 4C**), resulting in the release of more transcription factors (TF), which may further increase the production of the third CAR.
b. The second structural form is mainly to further optimize the synNotch receptor. When the synNotch receptor is combined with the corresponding tumor antigen, dual-signaling CARs (or two independent CARs) are formed (**Figure 4D**). In theory, CARs with this structure should have equivalent anti-tumor impact as tandem AND-gate CARs, but it complicates plasmid construction due to the addition of an extra set of intracellular elements of CAR.

### Triple scFvs (the In-Series Three-Input Cascade Circuit)

Williams et al. (26) constructed the more complex 3-input AND-gate CARs, which have three CARs, on the basis of the original AND-gate CARs, which have two CARs. They used the physical series circuit concept and introduced two separate synNotch receptors. These CARs are activated in a similar way to a cascade reaction. First, the combination of synNotch 1 receptor and the tumor antigen (a) induce the expression of synNotch 2 receptor. Subsequently, the expression of c-CAR is induced by the combination of synNotch 2 receptor and the tumor antigen (b). Finally, c-CAR binds to the tumor antigen (c) and the CAR-T cell is activated (26) (**Figure 2E**). *In vitro* experiments showed that (26) “the in-series three-input cascade circuit” not only improve the accuracy of CAR-T cells, but also enhance the activation and tumor-killing ability of CAR-T cells.

The activation of 3-input AND-gate CAR-T cells requires special conditions, which includes the simultaneous expression of three different tumor antigens by tumor cells. Therefore, this design greatly improves the accuracy of CAR-T cells. However, because four stages are involved in the activation of 3-input AND-gate CAR-T cells, it takes longer than other traditional CAR-T cells. Additionally, although this design improves the accuracy of CAR-T cells, it is ineffective against tumor cells that have lost the target antigen, which is a great disadvantage.

### Inhibitory CARs (iCARs)

**Figure 5:**
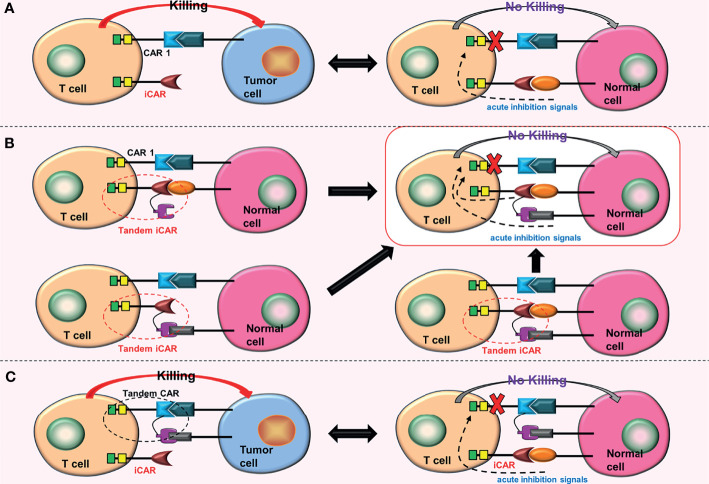
Some conjectures about the iCARs. **(A)** When iCAR and the non-tumor cell surface antigen are combined, acute inhibitory signals are produced, inhibiting the function of CAR 1. **(B)** Replacing the iCAR with a tandem iCAR. As long as one of the scFvs binds to the corresponding non-tumor cell surface antigen, acute inhibitory signals will be generated. These acute inhibitory signals will inhibit the function of the CAR 1. **(C)** Combining an iCAR with a TanCAR. The iCAR will also inhibit the function of the TanCAR after being activated.

The iCAR was constructed by Fedorov et al. (27) to distinguish between tumor cells and non-tumor cells and to suppress the T cell reaction once activated. There are two kinds of CARs on the surface of iCAR-T cells: traditional CARs, which target tumor cell surface antigens, and iCARs, which target non-tumor cell surface antigens. The iCAR contains a surface antigen recognition region for non-tumor cells and an acute inhibition signal region. CAR-T cells are activated when traditional CARs bind to the target tumor antigens, killing tumor cells. However, when iCARs bind to the target non-tumor antigens, they generate inhibitory signals that inactivate traditional CARs, protecting non-tumor cells from damage (**Figure 5A**). ICARs can effectively reduce the off-target effects of CAR-T cells. However, one drawback of this design is that finding related surface antigens, which are missing or downregulated in tumor tissues but highly expressed in non-tumor tissues, is difficult.

### Conjectures

The iCAR can be upgraded to tandem iCARs, which will enhance the recognition range of iCARs for non-tumor cell surface antigens and hence reduce the effects of off-target more effectively (**Figure 5B**). Furthermore, iCARs can be combined with tandem CARs to increase the range of tumor cell surface antigens recognized by CAR-T cells (**Figure 5C**).

### AND-NOT CARs

**Figure 6:**
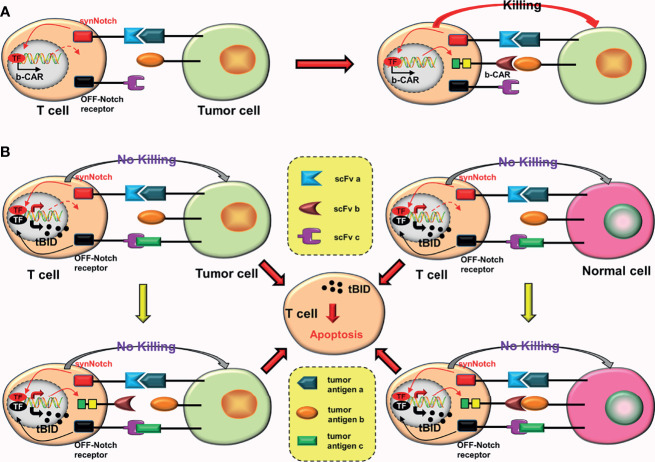
AND-NOT CARs. **(A)** The synNotch receptor binds to the corresponding tumor antigen (a), causing the expression of b-CAR genes. Subsequently, the newly expressed b-CAR binds to the target antigen (b). Finally, the CAR-T cell is activated. **(B)** The binding of the OFF-Notch receptor to the corresponding antigen (c) induces the expression of tBID, regardless of whether b-CAR is formed and whether it binds to the corresponding tumor antigen. Apoptosis of the CAR-T cell can be induced by the tBID.

To improve accuracy and tumor-killing ability of CAR-T cells while reducing toxic reactions, Williams et al. (26) constructed the OFF-Notch receptor by innovatively combining the “AND gate” with the “NOT gate”. Combining OFF-Notch receptors with the corresponding tumor antigens may promote the expression of proapoptotic factor truncated BH3-interacting domain death agonist (tBID), which may eventually induce CAR-T cells apoptosis (26). The mechanism of AND-NOT CARs is as follows: synNotch receptors bind to the corresponding tumor antigens (a), inducing the expression of b-CARs; subsequently, b-CARs bind to tumor antigens (b); and finally, CAR-T cells are activated (**Figure 6A**). However, as long as the OFF-Notch receptors bind to the corresponding antigens (c), the expression of tBID will be induced, and eventually cause apoptosis of the CAR-T cells (**Figure 6B**).

This design is equivalent to a complex version of iCARs. When compared to the iCARs, AND-NOT CARs improve accuracy (by adding the AND-gate system) and anti-tumor effect (double targets) of CAR-T cells. However, AND-NOT CAR-T cells face a significant challenge: OFF-Notch receptors must be timely activated to promptly promote apoptosis of these effector CAR-T cells.

## Improving the Killing Ability of CARs

### Special CARs Based on the Traditional Fourth-Generation CARs

The intracellular domain of traditional fourth-generation CARs have only one cytokine receptor. Adach et al. (28) constructed 7 × 19 CAR-T cells that expressed IL-7 and CCL19 chemokines synchronously. When compared to the traditional fourth-generation CAR-T cells, 7 × 19 CAR-T cells not only have stronger proliferation ability, endurance, and anti-tumor ability, but also improve the ability to recruit immune cells.

### Conjectures

**Figure 7:**
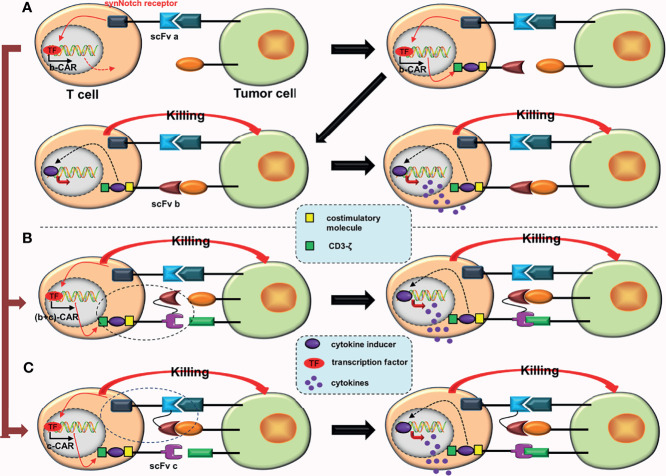
Some conjectures about special CARs based on the fourth-generation CARs. **(A)** The SynNotch receptor binds to the corresponding tumor antigen (a), inducing the expression of b-CAR, which is a fourth-generation CAR. When activated, the CAR-T cell will release extra cytokines. **(B)** The SynNotch receptor binds to the corresponding tumor antigen (a), which induces the expression of (b + c)-CAR (a tanCAR based on the fourth-generation CAR). When activated, the CAR-T cell releases extra cytokines. **(C)** The SynNotch receptor has two scFvs connected in tandem, and as long as one scFv binds to the corresponding tumor antigen, it will induce the expression of c-CAR (a fourth generation CAR); when activated, the CAR-T cell will also release extra cytokines.

a. As aforementioned, constructing the fourth-generation TanCARs (**Figures 1C, D**).
b. Based on AND-gate CARs, a cytokine inducer is added to the intracellular domain of the second CAR, which upgrades a second-generation CAR to a fourth-generation CAR (**Figure 7A**). Now that tandem AND-gate CARs (**Figure 3B**) can be successfully constructed, this design should also be feasible.
c. Based on tandem AND-gate CARs, a cytokine inducer is added to the intracellular domain of the second CAR, upgrading the second-generation CAR to a fourth-generation CAR (**Figures 7B, C**). Addition of a fourth-generation CAR may enhance the activation and anti-tumor effects of these CAR-T cells. Similarly, it is more difficult to construct these CAR-T cells.

### CARs That Can Reverse the Inhibition of Immune Checkpoints or Tumor Microenvironment

#### Chimeric-Switch Receptor (CSR)

**Figure 8:**
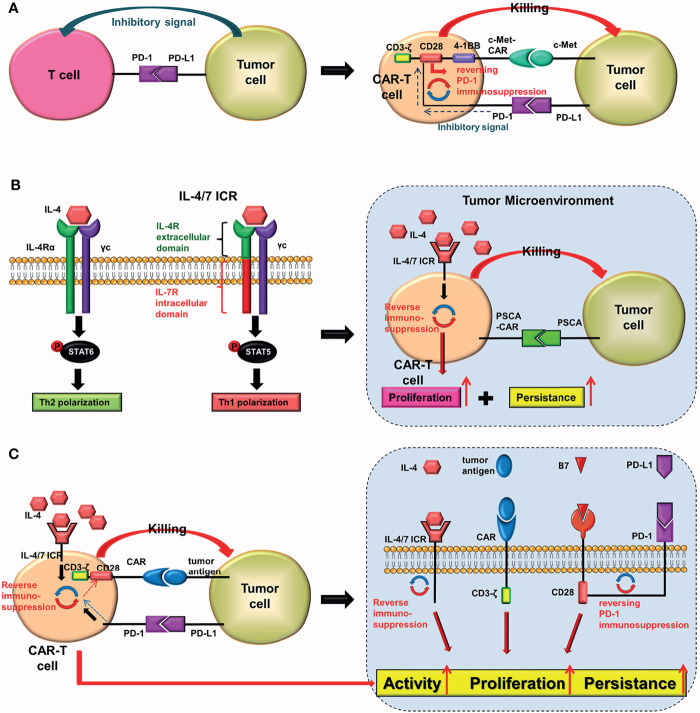
PD-1/CD28 CSR and IL-4/7 ICR. In T cells: **(A)** the PD-1/CD28 CSR converts inhibitory signals delivered by PD-1 into activation signals, whereas **(B)** the IL-4/7 ICR converts inhibitory signals delivered by IL-4 into activation signals. The IL-4/7ICR can improve the proliferation and persistence of CAR-T cells in an IL-4-rich tumor microenvironment. **(C)** The PD-1/CD28 CSR and the IL-4/7 ICR are both injected into CAR-T cells simultaneously.

Combining programmed cell death protein-1 (PD-1) on the surface of T cells with PD-L1 on the surface of tumor cells produces inhibitory signals that prevent the activation and proliferation of T cells. Prosser et al. (29) were the first to design a novel PD-1/CD28 chimeric-switch receptor (CSR), which could reverse (rather than block) the PD-1 immunosuppression. Chen et al. (30) combined CSR with the third-generation CAR (CD28, 4-1BB) to construct cMet-PD1/CD28-CAR-T cells for treating gastric cancer. In their experiment, they combined the extracellular structure of PD-1 with the transmembrane and intracellular domains of CD28 to form CSR. The PD-1/CD28 CSR could convert the immunosuppression transmitted by PD-1 into activation signals in cells (**Figure 8A**). Preclinical experiments revealed that cMet-PD1/CD28-CAR-T cells had higher anti-tumor effects and safety than traditional c-Met CAR-T cells (30).

Although the cMet-PD1/CD28-CAR-T cells belong to the third-generation CAR-T cells group in principle, they are better third-generation CAR-T cells. Despite the fact that the cMet-PD1/CD28-CAR has only one scFv, it can simultaneously have another target (PD-1). Therefore, the function and anti-tumor effect of this CAR may be equivalent to that of a TanCAR.

### Conjectures

a. Adding other immune checkpoints. More inhibitory signals can be reversed by combining the extracellular structures of two different immune checkpoints with intracellular CD28.
b. Replacing the intracellular 4-1BB of the cMet-PD1/CD28 CAR with a cytokine receptor (upgrading from the third-generation CAR to the fourth-generation CAR). Although the total number of components of this CAR remains unchanged when compared to cMet-PD1/CD28-CAR, this type of CAR-T cells can also release some anti-tumor cytokines.

#### Inverted Cytokine Receptor (ICR)

The tumor microenvironment (TME) is rich in IL-4. It has been proven that IL-4 promotes tumor growth while also protecting tumor cells from autoimmune destruction (31). To reverse the inhibitory effect of IL-4, Leen et al. (32) innovatively constructed the IL4/7 chimeric receptor (IL4/7 ChR), which is also known as IL4/7 inverted cytokine receptor (IL-4/7 ICR), by fusing the extracellular domain of the IL-4 receptor with the intracellular domain of the IL-7 receptor. The downstream signal generated by the combination of IL-4/7 ICR and IL-4 would eventually be sent out through the intracellular domain of the IL-7 receptor, where it would be converted into an activation signal. The results showed that IL-4/7 ICR could reverse the inhibitory effect of IL-4, and enhance the persistence and anti-tumor activity of T cells (maintaining Th1 phenotype). Mohammed et al. (33) used IL-4/7 ICR to construct CAR-T cells that target prostate stem cell antigen (PSCA) for treating pancreatic cancer. The preclinical study showed that these CAR-T cells could not only survive in IL-4-rich TME, but that IL-4 could also boost their activity and anti-tumor ability (**Figure 8B**). Wang et al. (34) constructed the IL-4/21 ICR-CAR-T cells, which were shown to be potentially safer than IL-4/7 ICR-CAR-T cells. Moreover, IL-4/21 ICR-CAR-T cells could only be activated when IL-4 and target antigen coexisted.

These two designs enable CAR-T cells to play a potent anti-tumor role in the IL-4-rich TME. Although both designs can reverse inhibitory signals, there are essential differences between them. The PD-1/CD28 CSR reverses inhibitory signals transmitted by tumor cells, whereas the IL-4/7 ICR reverses inhibitory effects of IL-4.

### Conjectures

Both the PD-1/CD28 CSR and the IL-4/7 ICR should be combined (**Figure 8C**). After being activated, CAR-T cells expressing these two receptors may be able to reverse the inhibitory signal transmitted by tumor cells as well as the inhibitory effect of IL-4 simultaneously (29, 32, 34–37).

### Universal CARs

**Figure 9:**
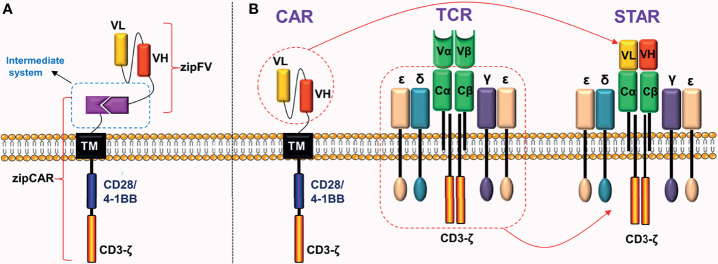
Universal CAR and STAR. **(A)** Adding a “third party” intermediate system to the extracellular domain of CAR. **(B)** STAR is mainly composed of two parts: the scFv of CAR and the constant region of TCR.

One of the biological hallmarks of malignant tumors is antigen heterogeneity. Because traditional CARs can only target one tumor antigen, it is possible for tumor cells that do not express or underexpress these antigens to elude the immune system during treatment. To improve the flexibility of CARs and expand the range of antigen recognition, researchers have developed universal CARs, also known as fifth-generation CARs. Universal CARs can overcome tumor antigen heterogeneity better than traditional CARs. Unlike traditional CARs, universal CARs have a “third party” intermediate system between the transmembrane domain and the scFv. Currently, universal CARs mainly include BBIR CAR and SUPRA CAR. As an example, the SUPRA CAR system consists of two parts: scFv with leucine zipper adaptor (zipFV) and T cell universal receptor with leucine zipper adaptor (zipCAR) (38). Combining zipFV and zipCAR will induce T cell activation (**Figure 9A**). Universal CARs are currently in the preclinical research stage.

### Synthetic T Cell Receptor and Antigen Receptor (STAR)

CAR-T cell therapy has shown a high response rate and lasting disease control in hematological malignancies. Although TCR is stronger than CAR in signal transduction (39), co-expression of the two on the surface of T cells is not recommended. One of the principles of producing universal T cells is to knockout TCR genes and HLA class I genes of T cells, preventing the occurrence of graft-versus-host response (GvHD). Although knocking out the TCR genes improves anti-tumor effects of CAR-T cells, it reduces their persistence (40).

To overcome these defects, Liu et al. (39) did not co-express TCR and CAR on the surface of T cells, but instead used another innovative idea to fuse their structures and successfully constructed a novel chimeric receptor. This is a double-chain chimeric receptor referred to as STAR. The STAR contains a specific scFv of the CAR, which recognizes the tumor antigen, and the constant region of TCR, which participates in endogenous signal transduction (**Figure 9B**). Therefore, STAR combines the advantages of the CAR and the TCR. Preclinical experiments revealed that (39) the anti-tumor ability of STAR-T cells in a variety of solid tumor models was obviously superior to traditional CAR-T cells. The advantages were mainly manifested in the fact that STAR further improved the antigen sensitivity, persistence, and proliferation ability of T cells without causing obvious toxic reactions.

## Improving the safety of CARs

If the proliferation of CAR-T cells is not controlled once they are inserted into patients, significant toxic reactions may occur. According to meta-analyses, the incidence of cytokine release syndrome (CRS) is about 55.3% (41), and the incidence of immune effector cell-associated neurotoxicity syndrome (ICANS) is approximately 37.2% (41) or 21.7% (42) in patients with hematological malignancies receiving CAR-T cell therapy. In recent years, certain non-traditional CARs that can reduce toxic reactions have emerged.

### ON/OFF-Switch CAR

**Figure 10:**
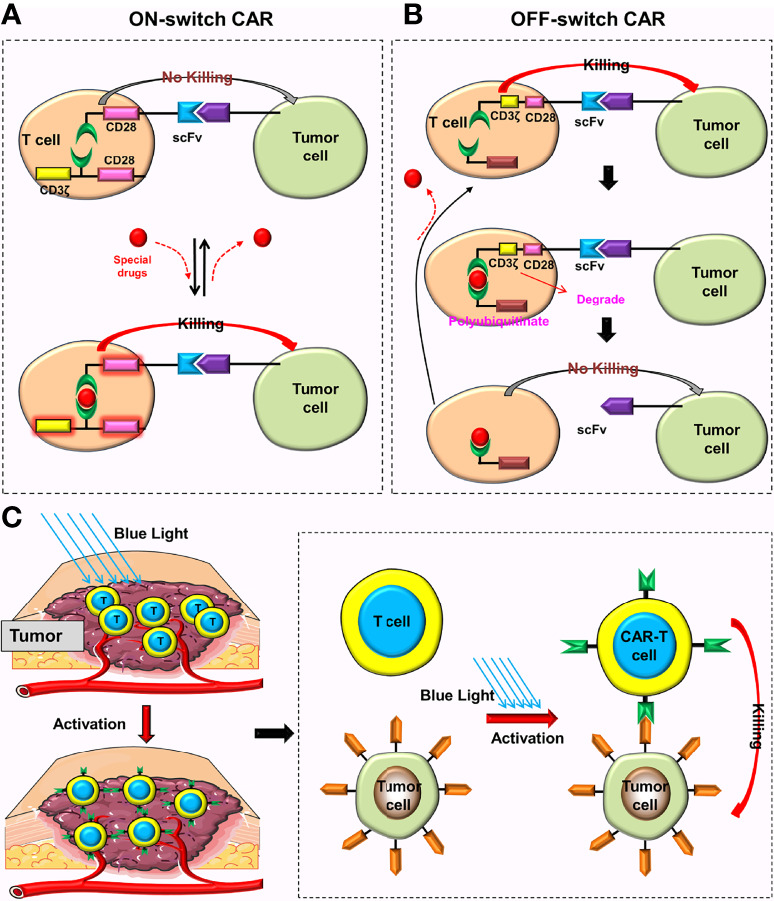
Special CARs that can reduce toxic reactions. **(A)** Only when special drugs exist can the ON-switch CAR return to its complete state and be activated. When these drugs are removed, the ON-switch CAR will gradually return to the inactive state. **(B)** In the absence of lenalidomide, the OFF-switch CAR-T cell can be normally activated. In the presence of lenalidomide, the labeled CAR proteins will be degraded, preventing the CAR-T cell from recognizing the tumor cell. CAR proteins will gradually be generated after these drugs are removed. **(C)** These CAR genes can only be activated and translated into CAR proteins under the irradiation of blue light.

To control the activation of CAR-T cells more accurately and prevent them from overreacting, Wu et al. (43) constructed a split synthetic receptor system. They used the system to divide the intracellular signal domain of a CAR into two parts (costimulatory domain and CD3ζ), and construct a special CAR called ON-switch CAR. ON-switch CAR-T cell is initially in an inactivated state, because the intracellular costimulatory domain and CD3ζ are in separate states. The intracellular costimulatory domain and CD3ζ can be reassembled and the intracellular domain restored to a complete state only with the use of a specially designed drug (rapamycin analog AP21967). Finally, the ON-switch CAR-T cell can be activated after combining with the corresponding tumor antigen (**Figure 10A**). Local administration of activating drugs can better reduce off-target effects for on-switch CAR-T cells.

Jan et al. (44) constructed the ON-switch CAR (Lenalidomide ON-switch split CAR) and the OFF-switch CAR (Lenalidomide OFF-switch degradable CAR). The ON-switch CAR uses the same design principle as Wu et al. (43), and a special drug is also needed to reassemble the intracellular domain of the CAR. The OFF-switch CAR uses “targeted protein degradation technology”, which could degrade the labeled CAR proteins in the presence of Lenalidomide, preventing CAR-T cells from recognizing tumor cells. After stopping using Lenalidomide, T cells produce new CAR proteins and gradually restore anti-tumor function (**Figure 10B**). This design can limit the short-term toxicity of CAR-T cells (the time depends on the metabolic time of Lenalidomide) but has no effect on their long-term anti-tumor efficacy.

### Conjectures

The aforementioned designs are mainly aimed at alleviating toxic reactions, and the next step should be to further improve their anti-tumor effects.

a. Constructing the ON/OFF-switch CAR based on a fourth-generation CAR.
b. Combining the ON/OFF-switch CARs with TanCARs to expand their recognition range of tumor antigens.

### CARs That Require Special Conditions (Light or Ultrasound) to be Activated

The light-switchable gene systems can regulate the expression of target genes by adjusting light intensity and duration (45). These systems not only realizes temporal and spatial control of gene expression, but they also improve the accuracy and safety of anti-tumor therapy, which has great potential in the treatment of malignant tumors (45–47). Huang et al. (48) constructed a light-inducible nuclear translocation and dimerization (LINTAD) system that enabled them to control the expression of CAR genes by blue light and the activation of CAR-T cells by regulating genes (**Figure 10C**). They found that light stimulation for 12 hours could achieve the maximum induction, and that this induction ability could last for about two days. Therefore, the activated CARs could only last for two days after turning off the illumination. With the help of the LINTAD system, the activation of CAR-T cells at the appropriate place and time can precisely be controlled, and be limited to the tumor site. The results showed that these CAR-T cells could minimize off-target toxicity and improve safety while ensuring anti-tumor activity (48). Additionally, Allen et al. (49) constructed the TamPA-Cre system, a novel genetic AND-gate switch that can induce the activation of CAR genes only when special drugs (tamoxifen) and blue light were simultaneously present. Their results revealed that the TamPA-Cre system could accurately control the local expression of CARs and subsequent activation of CAR-T cells while further reducing off-target toxicity (49).

Nevertheless, each of these designs has its own limitations. Due to the limited capacity to transmit blue light, these CAR-T cells are more suited to treat superficial tumors. Although red light or the red light system has a stronger penetration ability, it appears less effective than the blue light system (50), and requires additional auxiliary factors (51).

Ultrasound is a form of sound wave with a frequency higher than 20,000 Hz that can transport mechanical energy into the body (tens of centimeters from the body surface) safely and non-invasively (52, 53). On that account, the penetration ability of ultrasound far exceeds that of light (52, 53). Focused ultrasound (FUS) has thermal effects (54) that can make biological tissues to heat up locally. Wu et al. (55) constructed a heat-induced system, which was an eGFP reporter vector with a heat-shock proteins (Hsp) promoter. Additionally, they integrated Cre-lox gene switch into the system. Under heating conditions (43°C), T cells with this heat-induced system could express corresponding CAR proteins. In mice experiments, FUS was used to control the local temperature *in vivo* under the guidance of magnetic resonance imaging (MRI), and significant expression of CAR genes was observed in FUS-CAR-T cells with only two 5-minute FUS stimulation (Nalm-6 cells and double luciferase reporter gene were used to judge FUS-induced gene activation) (55).

To minimize the use of exogenous components, Wu et al, further optimized the FUS-CAR-T cells, in which the CARs expression was directly driven by Hsp without the use of a Cre-lox switch. Six hours after the first round of heat induction (43°C, 15 minutes), 43.9% of Hsp-CAR-T cells expressed CARs, and their expression level returned to the original basal level after 24 hours. During the second round of heat induction, 44.2% of Hsp-CAR-T cells expressed CARs, and the same degradation kinetics appeared. The experimental results showed that (55) Hsp-driven FUS-CAR-T cells were not only safe and effective, but also reversible.

### Conjectures

Huang et al. (48) also proposed several improved conjectures to enhance the limited penetration ability of blue light, including the use of up-conversion nanoparticles, which can convert near-infrared (NIR) light to stimulate blue light-responsive proteins, and implantable light-emitting diodes that were wirelessly controlled *via* radio frequency or NIR light for those solid tumors located deeper,.

### Adding Suicide Genes

Suicide genes are added to CAR-T cells (56, 57), and when the suicide genes are active, they induce irreversible apoptosis of CAR-T cells that cause toxic reactions or over-activation, thus reducing the toxic reaction. Common suicide genes include herpes simplex virus thymidine kinase (HSV-tk), the caspase 9 (iCasp9) suicide genes, and CD20 and truncated epidermal growth factor receptor (EGFRt). According to certain preclinical experiments, CAR-T cells containing suicide genes could reduce their toxic reactions and improve their safety (58–61). While the suicide genes regulate toxicity, they also inevitably trigger irreversible apoptosis of certain CAR-T cells, reducing the anti-tumor effect.

### Designing the Optimal Length of CD8α Hinge and Transmembrane Domain

The CD8α hinge and transmembrane domain play an important role in anti-tumor function and safety of CAR-T cells (62, 63). To reduce the adverse reactions of CAR-T cell therapy, Ying et al. (64) used the tertiary structure prediction program (Phrye2) to construct a group of CD19-BBz variants mainly by changing the length of CD8α hinge and transmembrane domain. Ying et al. found that CD19-BBZ (86) in this group of variants not only guaranteed the robust anti-tumor activity of CAR-T cells, but also significantly reduced their toxic reactions. Twenty-five patients with B lymphomas received CD19-BBZ (86)-CAR-T cells. The results of the study showed that anti-tumor effects of these CAR-T cells were not compromised, and the most encouraging thing was that none of the 25 patients had CRS (> Grade 1) or ICANS. Recently, Singh et al. (65) found that shortening the length of the linker can better activate CAR-T cells and improve their anti-tumor effects. Although the underlying mechanism is not clear, it is feasible to improve the anti-tumor activity and safety of CAR-T cells by optimizing the length of CD8α hinge, transmembrane domain, and linker.

## Summary

Chimeric antigen receptor-T (CAR-T) cell therapy has been proven to be a promising immunotherapy for hematological malignancies. Compared to hematological malignancies, CAR-T cells need to overcome more obstacles to play a better anti-tumor role in solid tumors. To improve the efficacy and safety of CAR-T cells in malignant tumors, numerous researchers are focusing on designing new CARs and optimizing the CAR framework structures (**Table 1**). Several new designs and optimizations of CARs have shown promising anti-tumor effects in preclinical and clinical trials. However, as the complexity of CAR framework structures and the increasing number of CAR components increases, constructing corresponding plasmids is becoming more difficult, and transfection efficiency is decreasing. Therefore, the concept of CAR construction in the future will still be to further streamline the components of CARs, optimize the structure of CARs, or build new CARs in order to ensure anti-tumor ability and safety. Some of the conjectures in this paper may be realized only partially or not at all. As gene-editing technology advances, we anticipate that these conjectures will be verified. Five CAR-T cell therapies have been approved for marketing so far, and we expect that an increasing number of malignant tumor patients will benefit from CAR-T cell therapy in the future.

**Table 1:** Summary of some CAR types.

Year	Authors	References	Journals	Types of CARs
2013	Grada et al.	TanCAR: A Novel Bispecific Chimeric Antigen Receptor for Cancer Immunotherapy (7)	Molecular Therapy Nucleic Acids	Tandem CAR (TanCAR)
2016	Ruella et al.	Dual CD19 and CD123 targeting prevents antigen-loss relapses after CD19-directed immunotherapies (15)	The Journal of Clinical Investigation	Dual-signaling CARs
2018	Bielamowicz et al.	Trivalent CAR T cells overcome interpatient antigenic variability in glioblastoma (16)	Neuro-oncology	Trivalent-tandem CAR
2016	Roybal et al.	Precision Tumor Recognition by T Cells With Combinatorial Antigen-Sensing Circuits (20)	Cell	synNotch AND-gate CAR
2020	Williams et al.	Precise T cell recognition programs designed by transcriptionally linking multiple receptors (26)	Science	Tandem AND-gate CAR
2020	Williams et al.	Precise T cell recognition programs designed by transcriptionally linking multiple receptors (26)	Science	3-input AND-gate CAR
2013	Fedorov et al.	PD-1- and CTLA-4-based inhibitory chimeric antigen receptors (iCARs) divert off-target immunotherapy responses (27)	Science Translational Medicine	Inhibitory CAR (iCAR)
2020	Williams et al.	Precise T cell recognition programs designed by transcriptionally linking multiple receptors (26)	Science	AND-NOT CARs
2018	Adach et al.	IL-7 and CCL19 expression in CAR-T cells improves immune cell infiltration and CAR-T cell survival in the tumor (28)	Nature Biotechnology	7 × 19 CAR (the fourth generation)
2012	Prosser et al.	Tumor PD-L1 co-stimulates primary human CD8(+) cytotoxic T cells modified to express a PD1:CD28 chimeric receptor (29)	Molecular Immunology	PD-1/CD28 CSR
2014	Leen et al.	Reversal of tumor immune inhibition using a chimeric cytokine receptor (32)	Molecular Therapy	IL-4/7 ICR
2019	Wang et al.	An IL-4/21 Inverted Cytokine Receptor Improving CAR-T Cell Potency in Immunosuppressive Solid-Tumor Microenvironment (34)	Frontiers In Immunology	IL-4/21 ICR
2018	Cho et al.	Universal Chimeric Antigen Receptors for Multiplexed and Logical Control of T Cell Responses (38)	Cell	Universal CAR
2021	Liu et al.	Chimeric STAR receptors using TCR machinery mediate robust responses against solid tumors (39)	Science Translational Medicine	STAR
2021	Jan et al.	Reversible ON- and OFF-switch chimeric antigen receptors controlled by lenalidomide (44)	Science Translational Medicine	ON/OFF-switch CAR
2020	Huang et al.	Engineering light-controllable CAR T cells for cancer immunotherapy (48)	Science Advances	Needing special light (LINTAD system)
2021	Wu et al.	Control of the activity of CAR-T cells within tumors *via* focused ultrasound (55)	Nature Biomedical Engineering	Needing focused ultrasound (FUS)
2019	Warda et al.	CML Hematopoietic Stem Cells Expressing IL1RAP Can Be Targeted by Chimeric Antigen Receptor-Engineered T Cells (58)	Cancer Research	Adding suicide genes
2019	Ying et al.	A safe and potent anti-CD19 CAR T cell therapy (64)	Nature Medicine	Designing the optimal length of CD8α hinge and transmembrane domain
2021	Singh et al.	Antigen-independent activation enhances the efficacy of 4-1BB-costimulated CD22 CAR T cells (65)	Nature Medicine	Optimizing the length of the linker

## Author Contributions

LM: Writing-Original draft preparation, manuscript, investigation, and figure preparation. JZ and BH: manuscript, investigation, and figure preparation. ZZ, SW, and FT: Investigation. MT: Investigation, methodology, supervision. YL: Conceptualization, methodology, supervision. All authors contributed to the article and approved the submitted version.

## Funding

This work was funded by Special Research Project of Lanzhou University Serving the Economic Social Development of Gansu Province (054000282) Lanzhou Talent Innovation and Entrepreneurship Project (2020-RC-38), the Fundamental Research Funds for the Central Universities (lzujbky-2020-kb14), and Major Science and Technology Special Project of Gansu Province (20ZD7FA003).

## Conflict of Interest

The authors declare that the research was conducted in the absence of any commercial or financial relationships that could be construed as a potential conflict of interest.

## Publisher’s Note

All claims expressed in this article are solely those of the authors and do not necessarily represent those of their affiliated organizations, or those of the publisher, the editors and the reviewers. Any product that may be evaluated in this article, or claim that may be made by its manufacturer, is not guaranteed or endorsed by the publisher.
